# Initial clinical evaluation of a novel integrative bone matrix (IBM) in foot and ankle fusion procedures

**DOI:** 10.1186/s12891-024-08110-9

**Published:** 2024-11-30

**Authors:** Kurt J. Hofmann, Nicholas J. Veale, Matt Veale, Evan Glass, Matthew Beckles

**Affiliations:** 1 Boston Sports & Shoulder Center, 840 Winter Street, Waltham, MA 02451 USA; 2 New England Baptist Hospital, Boston, MA 02120 USA

**Keywords:** Foot and ankle arthrodesis, Foot and ankle fusion, Demineralized bone graft, Integrative bone graft, Bone graft substitute, Concentrated bone marrow aspirate, cBMA

## Abstract

**Background:**

Foot and ankle arthrodesis procedures are frequently performed in concert with the utilization of bone grafts. However, the availability of autologous bone is often limited, inaccessible, or not suitable, thus there is a need for bone graft substitutes with equally effective clinical outcomes. A next generation integrative bone matrix (IBM) has been developed that has intrinsic osteogenic, osteoconductive, and osteoinductive characteristics, and is a promising solution to mitigate complications such as nonunion and reduce the need for autologous bone graft harvest.

**Methods:**

The charts of twenty subjects undergoing foot and ankle fusion procedures with INFLUX™ SPARC, a novel IBM, were retrospectively reviewed to determine initial clinical safety and efficacy of this next generation bone graft. Endpoints included the presence of complications or surgical reintervention, fusion rates as determined by standardized radiographic films, and patient-reported outcome measures at various time points up to 24 weeks.

**Results:**

No major complications or surgical reinterventions were observed in this study. Complete radiographic fusion was achieved in all subjects by 24 weeks, with a mean overall fusion time of 8.5 ± 4.8 weeks. Subjective pain, function, and quality of life outcomes showed consistent improvements throughout the follow-up period, and all subjects (100%) achieved the minimum clinically significant mean improvements for all measures by week 24.

**Conclusions:**

This study supports the use of this new IBM as a promising alternative to autologous bone grafting, offering high fusion success rates, low complications, and clinically meaningful improvements in patient-reported outcomes, particularly in higher-risk patient populations. Future investigations are needed to confirm these findings in larger and longer-term studies, and to explore the broader applications of this innovative bone graft.

## Background

Foot and ankle arthrodesis surgery can alleviate pain, enhance function, and improve quality of life for patients suffering from deformities, fractures, joint instability, injuries, trauma, or advanced arthritis when conservative treatments fail. However, nonunion at the fusion site is a prevalent complication, occurring in up to 41% of patients undergoing ankle, hindfoot, or midfoot arthrodesis [1, 2]. Nonunion can lead to increased pain, disability, prolonged recovery, and the need for additional surgical intervention(s). Preventing nonunion is crucial to improve clinical outcomes and reduce healthcare costs.

To prevent nonunion, surgeons typically supplement standard fixation with autologous bone harvested from either a local source (i.e. near the surgical site) or the iliac crest, as they contain essential osteogenic, osteoinductive, and osteoconductive components for rapid bone fusion [3, 4]. However, obtaining autologous bone can be challenging in foot and ankle procedures due to restrictions on iliac crest access, insufficient donor tissue, or patient conditions compromising donor tissue viability [3, 4]. Harvesting autologous bone also adds surgical time, a potential additional surgical site, and carries risks of complications such as donor site pain, scarring, and infection with reported rates as high as 5.9% [3–5].

As a result, allograft bone substitutes derived from donor tissue are frequently used to replace or reduce the reliance on autograft due to their reported safety profile and equivalent fusion rates [3]. The latest advancement in allograft technology includes allogenic cellular bone matrices (CBM), which retain the native scaffold, osteoinductive growth factors, and viable bone-forming cells. However, the processing steps used during CBM manufacturing can remove essential native growth factors crucial for angiogenesis and cell proliferation, which could impact the graft’s effectiveness. Also, reports of variability in cell and osteoinductive protein concentrations between allogenic bone grafts (and sometimes between their manufactured lots) can lead to difficulty in assessment of their performance and predictability [6]. Since angiogenesis is vital for nutrient and cell transportation during bone healing, there is a demand for advanced cellular allografts that better conserve these growth factors to enhance bone formation [7]. 

A next generation integrative bone matrix (IBM) was recently developed to preserve higher concentrations of native osteoinductive, chemotactic, angiogenic, and proliferative growth factors within the matrix compared to competitive CBM. This retrospective series reports our initial clinical experience from utilization of this new IBM in subjects who underwent foot and/or ankle arthrodesis.

## Methods

The charts of 20 consecutive subjects who received INFLUX™ SPARC Integrative Bone Matrix (IBM) (ISTO Biologics, Hopkinton, MA) along with standard fixation during their forefoot, midfoot, hindfoot, and/or ankle fusion from a single surgeon (KJH) between October 2022 and January 2023 were retrospectively reviewed to assess initial clinical safety and efficacy of the graft. Each cohort received the same postoperative protocol: following forefoot and midfoot fusion procedures, patients were allowed to partially weight bear in a medical boot with a dressing to keep the incisions dry and clean. Two weeks postoperatively, patients returned to the clinic for non-weight bearing radiographs and suture removal. At this point, they remained partial weight bearing in the medical boot. Six weeks postoperatively, patients were assessed with weight bearing radiographs and typically began physical therapy. Patients progressed to full weight bearing while wearing the medical boot. By twelve weeks postoperatively, patients returned for additional weight bearing radiographs and were generally advanced to full weight bearing without the medical boot. Immediately following hindfoot and ankle fusion surgical procedures, patients remained non-weight bearing in a postoperative splint. Two weeks postoperatively, they returned to the clinic for splint and suture removal, along with non-weight bearing radiographs. They continued to be non-weight bearing in a medical boot or cast until the six-week visit. At that time, patients received additional non-weight bearing radiographs and typically progressed to touchdown weight bearing in a medical boot. By their twelve-week postoperative visit, patients had weight bearing radiographs and progressed to weight bearing as tolerated in the medical boot. Finally, at the twenty-four-week postoperative visit, patients received additional weight bearing radiographs and had progressed to full weight bearing.

The primary endpoint was the incidence of revision surgery. The secondary endpoint was radiographic fusion as determined by the operative surgeon (KJH) through plain radiographs that were blinded from patient identifiers, but not follow up time. Exploratory endpoints included multiple patient-reported outcome measures collected per the operative surgeon’s standard of practice. The local IRB granted a waiver of informed consent and HIPAA authorization due to the retrospective nature of this study.

Standardized radiographic evaluation of anteroposterior, oblique, and lateral radiographic views were performed at each follow up time point (2-, 6-, 12-, and 24-weeks) and compared to pre-operative (baseline) radiographs to evaluate fusion rate. A subject was deemed “fused” by the operative surgeon if 50% or more of their fusion site exhibited osseous bridging at each postoperative radiographic evaluation [8, 9]. 

Subjects were also evaluated for pain, function, and quality-of-life via patient-reported outcome measures (PROMs) at baseline (preoperative), and 2-, 6-, 12-, and 24-weeks. To evaluate pain, a Visual Analog Scale (VAS) was used which ranged from 0 points (no pain) to 10 points (worst pain imaginable). Both the Foot Function Index (FFI) [10] total score (0 points for best to 100 points for worst results) and the American Orthopaedic Foot and Ankle Society (AOFAS) Score [11] (three categories of patient and surgeon reported scores [pain, alignment, function] from 0 points for worst to 100 points for best results) were used to evaluate function, however it was not our standard of practice to collect these two outcome measures at the 2-week follow up timepoint. Quality-of-life with both a physical component score (PCS) and mental component score (MCS) was evaluated using SF-12, ranging from 0 points for worse to 100 points for better physical and mental health functioning [12]. 

The cohort demographics and clinical outcomes were described using means with standard deviations, medians with minimum and maximum scores, and counts with percentages. Fusion rates at each follow up timepoint and time-to-fusion were compared between sub-cohorts using Fisher’s exact test and independent samples t-test, respectively. Postoperative improvements in PROMs (Pain VAS, FFI, AOFAS, SF-12 MCS, and SF-12 PCS) compared to baseline were analyzed using paired samples t-tests. The alpha-risk was set to 0.05 for all statistical comparisons performed. R statistical software was used for analyses (version 4.2.2, R Foundation for Statistical Computing, Vienna, Austria).

## Results

Interventions performed on 20 total subjects included joint fusions and repair of fracture nonunion in the forefoot/midfoot (*n* = 10) and hindfoot/ankle (*n* = 10) regions. Minor bone defects were present in several cases; specifically, these included one patient with a Lisfranc injury, a patient with osteochondral lesions of the medial talar dome, a patient with stress fractures of the lateral navicular and talus, and another patient with osseous calcaneonavicular coalition. None of these cases demonstrated substantial preoperative defects or severe malalignment. The total patient population was 50% male with a mean age of 62.2 ± 9.3 years (range 38–76) and mean BMI of 29.6 ± 6.0, however the hindfoot/ankle cohort was numerically younger than the forefoot/midfoot cohort (58.0 ± 10.6 vs. 66.3 ± 5.6, respectively), more male dominant (70.0% vs. 30.0%, respectively), and had a lower frequency of obesity (30.0% vs. 50.0%, respectively) (Table 1). Most subjects (85%) had 1 or more existing comorbidities, 40% were current or former smokers, and 10% underwent revision repairs of previous surgeries (Table 1). The mean American Society of Anesthesiologists (ASA) physical status classification score was 2.1 ± 0.4 (Tables 1 and 2) [13]. 

**Table 1:** Patient demographics & clinical characteristics

	Total Cohort (*n* = 20)	Forefoot/Midfoot (*n* = 10)	Hindfoot/Ankle (*n* = 10)
**Age**			
Years, mean ± SD	62.2 ± 9.3	66.3 ± 5.6	58.0 ± 10.6
(Min – Max)	38–76	57–76	38–69
**Gender**			
Male	10 (50.0%)	3 (30.0%)	7 (70.0%)
**BMI**			
kg/m^2^, mean ± SD	29.6 ± 6.0	31.2 ± 7.2	28.0 ± 4.2
**Race**			
White	18 (90.0%)	9 (90.0%)	9 (90.0%)
Other	2 (10.0%)	1 (10.0%)	1 (10.0%)
**Ethnicity**			
Non-Hispanic or Latino	19 (95.0%)	10 (100.0%)	9 (90.0%)
Hispanic or Latino	1 (5.0%)	0 (0.0%)	1 (10.0%)
**Smoker**			
Current	3 (15.0%)	1 (10.0%)	2 (20.0%)
Former	5 (25.0%)	2 (20.0%)	3 (30.0%)
**Diagnoses at enrollment**			
None	3 (15.0%)	1 (10.0%)	2 (20.0%)
Obesity (> 30 BMI)	8 (40.0%)	5 (50.0%)	3 (30.0%)
Hypothyroidism	5 (25.0%)	3 (30.0%)	2 (20.0%)
Hypercholesteremia	4 (20.0%)	2 (20.0%)	2 (20.0%)
Hypertension	8 (40.0%)	4 (40.0%)	4 (40.0%)
**ASA Score (1–5)**			
Mean ± SD	2.1 ± 0.4	2.0 ± 0.5	2.2 ± 0.4
**Previous Surgeries**			
Yes	2 (10.0%)	0 (0.0%)	2 (20.0%)

ASA: American Society of Anesthesiologists; BMI: Body Mass Index; SD: Standard Deviation

**Table 2:** American Society of Anesthesiologists (ASA) physical status classification

**I**	A normal healthy patient	
**II**	A patient with mild systemic disease	
**III**	A patient with severe systemic disease	
**IV**	A patient with severe systemic disease that is a constant threat to life	
**V**	A moribund patient who is not expected to survive without the operation	

Preparation of the IBM graft during each procedure was performed per manufacturer instructions, and all bone graft constructs consisted of at least 50% IBM. Other bone graft components that were used included autologous concentrated bone marrow aspirate (Magellan cBMA, ISTO Biologics, Hopkinton, MA) in a 1:1 ratio, and local bone or crushed cancellous allograft if needed to extend the graft (Table 3).

**Table 3:** Procedural details

	Total Cohort (*n* = 20)	Forefoot/Midfoot (*n* = 10)	Hindfoot/Ankle (*n* = 10)
**Total Amount of IBM Used**			
cc, mean ± SD	4.2 ± 3.7	1.6 ± 0.5	7.1 ± 3.6
**Other Materials Used with IBM** n (%) (could not be more than 50% of total graft volume)			
Autologous concentrated bone marrow	20 (100.0%)	10 (100.0%)	10 (100.0%)
Allograft cancellous bone	2 (10.0%)	0 (0.0%)	2 (20.0%)
Autologous bone	2 (10.0%)	0 (0.0%)	2 (20.0%)

IBM: Integrative Bone Matrix; SD: Standard Deviation

**Fig. 1:**
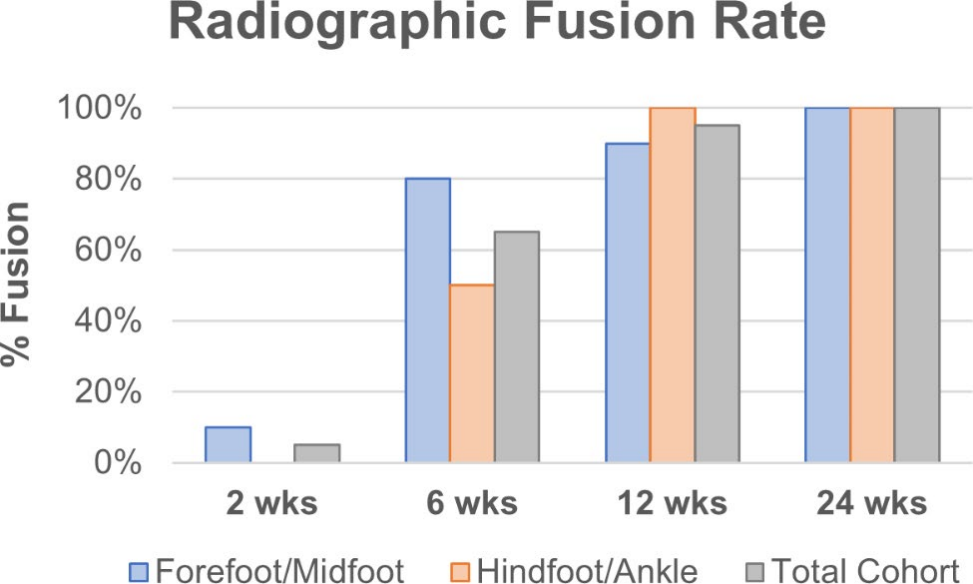
Radiographic fusion by cohort over time. Subjects were considered fused if ≥ 50% of the fusion site exhibited osseous bridging per operative surgeon evaluation. All subjects (100%) were fused by the 24-week evaluation

There were zero (0%) incidences of major perioperative/postoperative adverse events, or surgical reintervention due to graft failure or by any other means. There were 3 minor complications: 1 broken staple, 1 wound dehiscence, and 1 stable small broken limb of the 3rd tarsometatarsal joint, but all resolved without the need for additional surgical intervention. Despite some patients having minor bone irregularities, these subjects exhibited strong clinical outcomes and high union rates. By 6 weeks postoperative, radiographic evaluation revealed a 65% overall fusion rate (80% of forefoot/midfoot and 50% of hindfoot/ankle subjects; *p* = .350). At 12-weeks, there was a 95% overall fusion rate (90% of forefoot/midfoot and 100% of hindfoot/ankle subjects; *p* > .999). By the 24-week evaluation, all subjects (100%) were fused according to their radiographic outcomes (Table 4; Fig. 1). Overall mean time to fusion was 8.5 ± 4.8 weeks, with no statistically significant difference between forefoot/midfoot and hindfoot/ankle cohorts (8.0 ± 6.1 and 9.0 ± 3.2, respectively; *p* = .706).

**Table 4:** Radiographic fusion rates

	Total Cohort (*n* = 20)	Forefoot/Midfoot (*n* = 10)	Hindfoot/Ankle (*n* = 10)	*p*-value*
**Fusion Rate**, n (%)				
2 weeks	1 (5.0%)	1 (10.0%)	0 (0.0%)	*> 0.999*
6 weeks	13 (65.0%)	8 (80.0%)	5 (50.0%)	*0.350*
12 weeks	19 (95.0%)	9 (90.0%)	10 (100.0%)	*> 0.999*
24 weeks	20 (100.0%)	10 (100.0%)	10 (100.0%)	*> 0.999*
**Time to Fusion**, mean ± SD				
	8.5 ± 4.8	8.0 ± 6.1	9.0 ± 3.2	*0.706*

*p-value represents Forefoot/Midfoot versus Hindfoot/Ankle fusion rates at each follow up timepoint

**Fig. 2:**
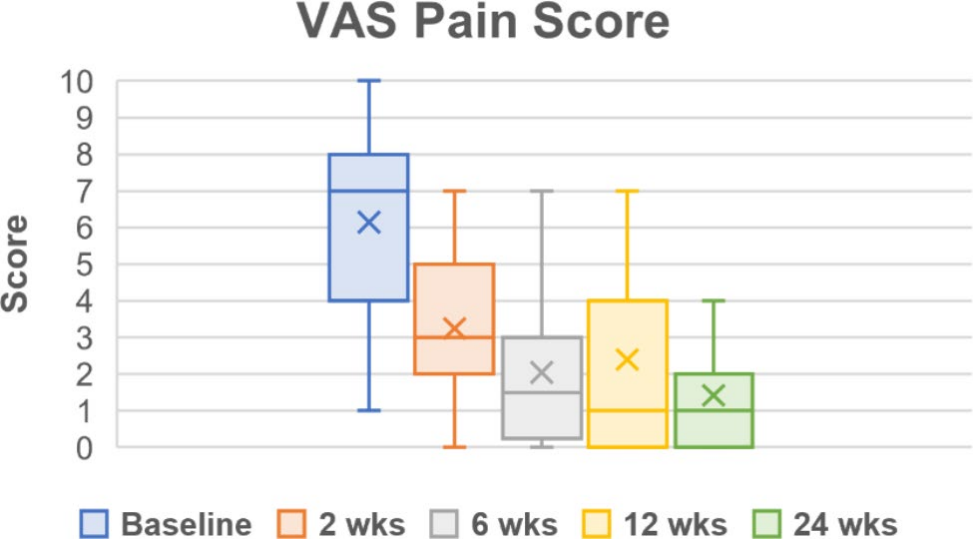
Visual Analog Scale (VAS) Scores for pain. Scores are presented as Median (IQR, 25th – 75th percentile) as reported in Table 5. Mean scores at each follow up timepoint were: 6.2 ± 2.4 (baseline), 3.3 ± 2.0 (2-week), 2.1 ± 2.0 (6-week), 2.4 ± 2.5 (12-week), and 1.4 ± 1.3 (24-week)

**Fig. 3:**
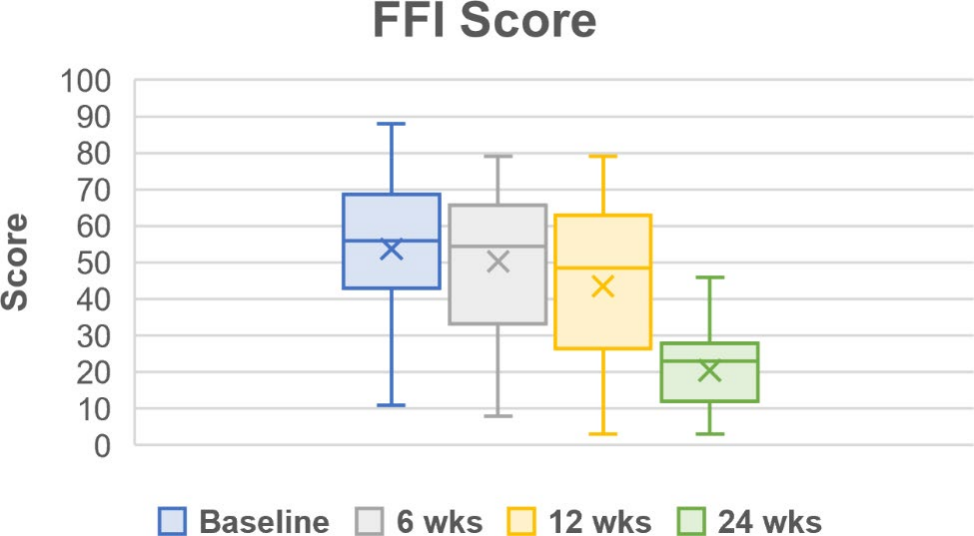
Foot Function Index (FFI) Scores for pain, disability, and activity restriction. Scores are presented as Median (IQR, 25th – 75th percentile) as reported in Table 5. Mean scores at each follow up timepoint were: 53.7 ± 21.6 (baseline), 50.4 ± 19.5 (6-week), 43.5 ± 22.3 (12-week), and 20.5 ± 13.0 (24-week)

**Fig. 4:**
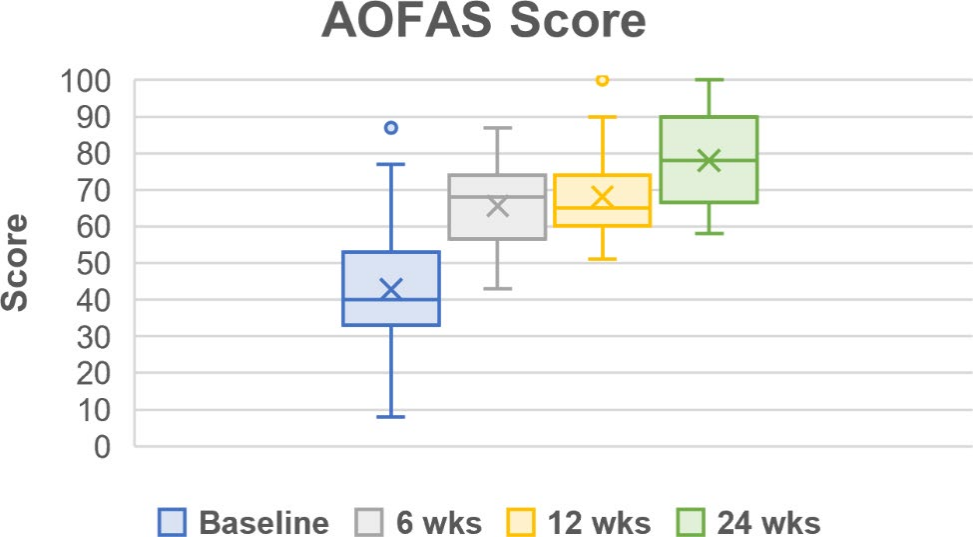
American Orthopaedic Foot and Ankle Society (AOFAS) Score for pain, alignment, and function. Scores are presented as Median (IQR, 25th – 75th percentile) as reported in Table 5. Mean scores at each follow up timepoint were: 42.8 ± 19.1 (baseline), 65.7 ± 11.6 (6-week), 68.2 ± 12.1 (12-week), and 78.1 ± 13.0 (24-week)

**Fig. 5:**
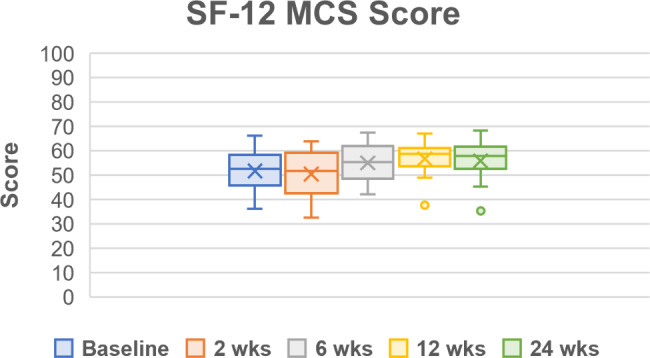
Short Form-12 (SF-12) for mental quality of life (MCS). Scores are presented as Median (IQR, 25th – 75th percentile) as reported in Table 5. Mean scores at each follow up timepoint were: 51.8 ± 8.9 (baseline), 50.5 ± 9.2 (2-week), 55.1 ± 8.3 (6-week), 56.8 ± 6.4 (12-week), and 55.9 ± 9.2 (24-week)

**Fig. 6:**
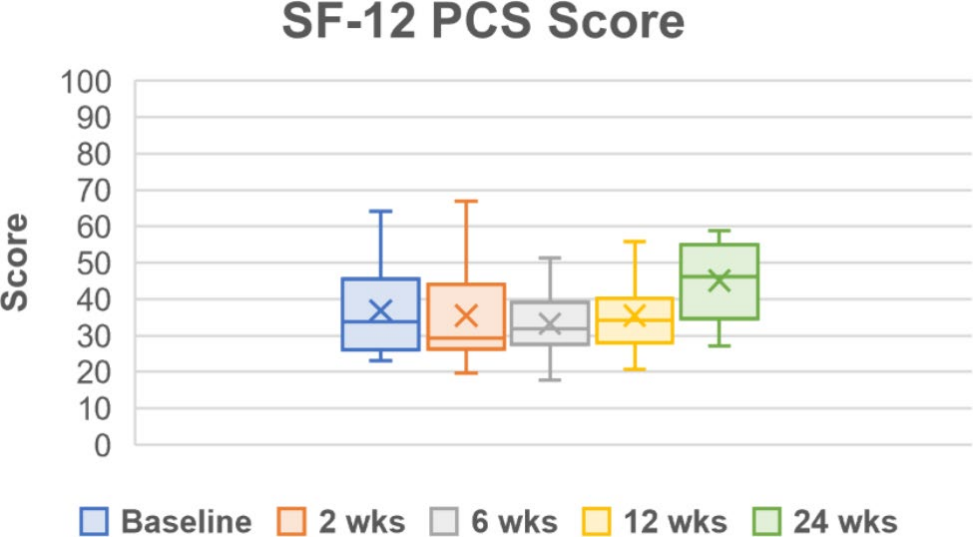
Short Form-12 (SF-12) for physical quality of life (PCS). Scores are presented as Median (IQR, 25th – 75th percentile) as reported in Table 5. Mean scores at each follow up timepoint were: 36.9 ± 12.6 (baseline), 35.4 ± 13.7 (2-week), 33.2 ± 8.5 (6-week), 35.4 ± 9.8 (12-week), and 45.1 ± 10.4 (24-week)

Throughout the follow up period, all PROMs showed improvements in pain, function, and quality of life (Table 5; Figs. 2, 3, 4, 5 and 6). One subject missed their 24-week AOFAS survey and radiograph, and another subject missed all their 24-week follow-up evaluations. However, both subjects were already graded as fused at 6 weeks postoperative. The mean change in reported VAS pain scores for the total cohort was − 4.8, which is an overall 77.4% decrease (improvement) over the 24-week follow-up period (*p* < .*001* vs. baseline) (Table 5; Fig. 2). Subject-reported data represents a composite FFI score, which decreased (improved) 61.8% over the 24-week follow-up period, equating to a 33.2-point improvement in mean score (*p* < .*001* vs. baseline) (Table 5; Fig. 3). Overall AOFAS scores increased (improved) by a mean score change of + 35.3 (82.5%) over the 24-week follow-up period (*p* < .*001* vs. baseline) (Table 5; Fig. 4). Subject reported SF-12 scores increased (improved) by an overall mean score change of + 4.1 (7.9%) for mental (MCS) and + 8.2 (22.2%) for physical (PCS) quality of life over the 24-week follow-up period (MCS: *p* = .103, PCS: *p* = .029 vs. baseline) (Table 5; Figs. 5 and 6). The greatest improvement for all scores compared to baseline was observed at the 24-week assessment: both individual cohorts and the total cohort (100%) displayed the minimum clinically significant mean improvement in VAS-pain (-2 points), FFI (-11 points), AOFAS (+ 20 points), and SF-12 MCS/PCS (+ 2 points) [8, 14].

**Table 5:** Pain and disability scores

	Total Cohort (*n* = 20)	*p*-value*	Forefoot/Midfoot (*n* = 10)	Hindfoot/Ankle (*n* = 10)
**Pain VAS** median (IQR, 25th – 75th %)				
Baseline	7.0 (4.0–8.0)	--	6.5 (4.3–7.0)	7.0 (4.5–8.0)
2 weeks	3.0 (2.0–5.0)	***0.002***	2.5 (2.0–3.8)	3.5 (2.0–5.0)
6 weeks	1.5 (0.8–3.0)	***< 0.001***	2.5 (1.3–3.0)	1.0 (0.3–1.8)
12 weeks	1.0 (0.0–4.0)	***< 0.001***	2.0 (0.0–4.0)	1.0 (1.0–3.8)
24 weeks	1.0 (0.5–2.0)	***< 0.001***	1.0 (0.3–1.0)	2.0 (1.0–2.0)
**Composite FFI** median (IQR, 25th – 75th %)				
Baseline	56.0 (45.0–68.3)	--	51.5 (33.0–65.5)	64.0 (49.5–70.8)
6 weeks	54.5 (35.8–65.3)	*0.511*	38.0 (26.0–55.0)	60.5 (53.8–66.8)
12 weeks	48.5 (27.5–61.0)	***0.048***	27.0 (14.0–41.5)	57.0 (51.8–65.5)
24 weeks	23.0 (12.0–28.0)	***< 0.001***	12.0 (3.8–23.8)	23.0 (18.0–30.0)
**AOFAS** median (IQR, 25th – 75th %)				
Baseline	40.0 (33.0–51.0)	--	52.0 (41.8–62.3)	33.5 (23.8–38.3)
6 weeks	68.0 (57.5–74.0)	***< 0.001***	74.0 (70.5–74.8)	58.0 (56.0–65.5)
12 weeks	65.0 (60.8–74.0)	***< 0.001***	74.0 (72.0–79.3)	60.5 (57.8–61.8)
24 weeks	78.0 (67.5–88.0)	***< 0.001***	90.0 (80.0–97.0)	70.0 (64.0–76.0)
**SF-12 - MCS** median (IQR, 25th – 75th %)				
Baseline	52.7 (46.2–58.2)	--	52.7 (47.1–56.4)	53.4 (46.2–58.2)
2 weeks	51.7 (43.9–58.6)	*0.634*	52.9 (41.1–58.7)	51.7 (46.6–56.9)
6 weeks	55.4 (50.5–61.5)	*0.249*	55.6 (51.5–61.3)	53.9 (48.7–60.0)
12 weeks	58.9 (54.6–61.2)	***0.069***	58.2 (53.9–59.1)	60.4 (55.1–61.7)
24 weeks	57.9 (53.5–61.6)	*0.103*	56.9 (53.2–60.7)	58.8 (54.2–64.4)
**SF-12 - PCS** median (IQR, 25th – 75th %)				
Baseline	33.8 (26.5–44.9)	--	38.5 (31.8–47.7)	27.2 (24.7–36.3)
2 weeks	29.4 (26.9–42.5)	*0.719*	37.1 (29.3–48.5)	28.1 (26.1–29.9)
6 weeks	32.0 (28.5–37.8)	*0.292*	35.0 (30.7–44.0)	30.7 (25.3–33.1)
12 weeks	34.2 (29.9–39.8)	*0.661*	38.2 (31.6–49.1)	33.6 (28.1–34.2)
24 weeks	46.1 (35.4–54.8)	***0.029***	54.3 (49.6–55.2)	34.7 (31.2–44.5)

AOFAS: American Orthopaedic Foot and Ankle Society; FFI: Foot Function Index; IQR: interquartile range; SF-12: Short Form-12; MCS: Mental Component Score; PCS: Physical Component Score; VAS: Visual Analog Scale

*p-value represents total cohort baseline scores versus scores at each follow up timepoint

## Discussion

The observed high fusion rates and low occurrence of complications from using this novel IBM suggests a superior safety profile compared to iliac crest autograft and aligns with reported high fusion rates and safety profiles of other bone graft substitutes [3–5]. Diminished blood supply at fusion sites or medical comorbidities may contribute to nonunion, suggesting that biologic grafts which promote angiogenesis could improve fusion rates. Preserved native growth factors and cells in the IBM may enhance neovascularization, encourage preosteoblast development, and counter wound healing issues associated with comorbidities [15]. 

In foot and ankle arthrodesis, nonunion rates are elevated, particularly among males, those with a high body mass index (BMI), and current smokers (*p* < .*05*) [1, 2, 16]. Supporting fusion and preventing nonunion are vital for improving clinical outcomes and cost reduction. This study cohort was relatively high-risk, with 50.0% male, 80.0% overweight or obese, and 40.0% current or former smokers. Despite this, our cohort had a 0% rate of major complications including nonunion, notably lower than reported nonunion rates of 10 – 41% in foot and ankle fusion procedures [1, 2]. No significant differences were found in fusion rates among smokers, those with higher ASA scores, obese patients, or those undergoing revision procedures. The subject with the longest time to achieve fusion (24 weeks) was relatively low risk, however 2 months post procedure they experienced a minor complication (broken staple at the medial cuneiform), which was treated conservatively, but this incident may have prolonged their time to fusion.

Demineralized bone matrix (DBM) is a common bone allograft containing growth factors and proteins crucial for bone healing. However, DBM often requires supplementation with exogenous cells or requires potent enough properties to effectively recruit endogenous cells essential for bone healing. In a study of Grafton DBM Putty (Medtronic, Minneapolis, MN), 14% of 37 subjects undergoing hindfoot and/or ankle fusions experienced nonunion after 1 year [17]. Another study comparing DBM (DBX inject, Depuy Synthes, West Chester, PA) to fixation alone in primary ankle arthrodesis showed similar rates of nonunion (12.9% with DBM vs. 14.8% without, *p* = .83) and return to surgery (29% with DBM vs. 37% without, *p* = .20) after a mean follow-up of 43 months [18]. Another DBM bone graft (OsteoAMP, Bioventus, Durham, NC) reportedly preserves essential growth factors better, potentially enhancing osteoinduction and angiogenesis. In a retrospective comparison study of 68 subjects undergoing ankle and hindfoot fusions, 29 subjects received this graft while 39 subjects received fixation alone or another bone graft. Results showed a mean time to fusion of 112.6 ± 74.4 days (almost double our observed mean), a nonunion rate of 10.3%, and a complication rate of 13.8% [19].

A synthetic graft (Augment, Wright Medical, Memphis, TN) combines beta-tricalcium phosphate (β-TCP)-collagen with recombinant human platelet-derived growth factor BB homodimer (rhPDGF-BB) to promote angiogenesis. A propensity score analysis of 132 subjects from three randomized ankle and hindfoot fusion studies showed comparable outcomes to iliac crest autograft (ICBG): fusion rates of 82.9% and 84.8% per patient at 24 and 52 weeks (compared to 88.5% and 90.7% for ICBG, respectively), a nonunion rate of 11.4% (7.8% for ICBG), and a device-related adverse event rate of 2.3% (3.6% for ICBG) [9]. These next generation osteoinductive DBM and synthetic grafts lack a cellular component, prompting the development of viable cellular bone allografts (CBM or VBM) and IBM to address the need for osteogenesis. In our study with the novel IBM, there were no observed instances of nonunion, and radiographic fusion was achieved in all subjects within 24 weeks, with an average fusion time of 8.5 ± 4.8 weeks (59.5 ± 33.6 days). These results are consistent with fusion rates reported in previous studies for both autograft and allograft in foot and ankle fusion procedures [9, 19–28].

Various manufacturing processes and methods have resulted in a wide range of cells and growth factors preserved within individual CBM products [29]. Foot and ankle arthrodesis studies using CBM report fusion rates ranging from 57–90% at 3 months, 69–100% at 6 months, and 71–93% at 12 months, with a mean time to fusion of 10.6 to 13.5 weeks and complication rates of 6.5–35% [22–28]. These findings are consistent with our results, except for the lower mean time to fusion (8.5 ± 4.8 weeks) and major complication rate (0%) in our study, possibly indicating the value of higher inductive factors in the presence of cells with IBM. The limited number of foot and ankle CBM studies assessing postoperative patient-reported outcome scores compared to baseline showed improvements in visual analog scale (VAS) and American Orthopaedic Foot & Ankle Society (AOFAS) scores, which align with our findings: a mean VAS improvement of 84.3% at 6 months post-operative (aligning with our finding of 83.3%) and 6, 12, and 24 weeks post-operative VAS improvements of 4.1, 3.6, and 3.4 points, respectively (aligning with our improvements of 3.0, 5.0, and 5.0 points), and a mean AOFAS improvement at 6, 12, and 24 weeks of + 17.4, + 23.9, and + 31.1 points, respectively (aligning with our improvements of + 27.0, + 23.0, and + 33.0 points) [23, 27].

The novel IBM utilized in this study is composed of cortical DBM fibers and cancellous chips, which offers osteoconduction, preserves bioavailable inductive proteins and viable osteoprogenitor cells, and has superior handling compared to particulate DBM in this author’s opinion [30]. Unlike traditional CBM, its proprietary processing method retains elevated levels of native growth factors that support the wound healing process, including various bone morphogenetic proteins (BMPs), platelet-derived growth factor (PDGF), transforming growth factor-β3 (TGF-β3), vascular endothelial growth factor (VEGF), and endothelial growth factor (EGF). Since different cytokines are active during various stages of bone healing, it is critical to utilize a graft that supports the entire microenvironment of fusion healing. It comes in a ready-to-use syringe with a DMSO-free cryoprotectant, requiring only a ~ 10-minute thaw in sterile water or saline at room temperature. The cells remain viable for up to 5 h post-thaw, eliminating the need for washing or decanting before use.

This is the first reporting of foot and ankle arthrodesis procedures utilizing this novel IBM, including both primary and revision surgeries across various joints. Fusion rates remained consistent regardless of primary or revision status, defying the usual trend of inferior outcomes in revision procedures. Notably, at-risk patients, including those with diabetes, obesity, or advanced age, achieved fusion rates comparable to lower-risk individuals. These findings underscore the suitability of this novel IBM not only for primary and revision procedures but also for higher-risk cases characterized by diverse procedural types, patient demographics, and medical comorbidities.

## Conclusions

Recent advancements in bone graft technology, such as IBM, have emphasized the presence of essential growth factors in the matrix to enhance bone healing and improve clinical union rates. This retrospective study underscores the promising safety and effectiveness of employing INFLUX™ SPARC Integrative Bone Matrix (IBM) across various fusion procedures in the foot and ankle, which led to high fusion success rates, minimal complications, and clinically meaningful improvements in patient-reported outcomes, particularly in a higher-risk population. The use of this novel IBM presents a feasible alternative to autograft, eliminating the need for an additional surgical site and associated complications of autograft bone harvest. Limitations to the study include its size and retrospective nature, the use of additional bone grafts, and radiographic evaluation by the operative physician. Larger studies with independent radiographic evaluation are necessary to validate these preliminary results and to further explore the clinical advantages of employing this IBM in diverse bone fusion applications.

## Data Availability

The dataset supporting the conclusions of this article is available upon reasonable request to the corresponding author.

## Abbreviations

AOFAS: American Orthopaedic Foot and Ankle Society
ASA: American Society of Anesthesiologists
β-TCP: Beta-Tricalcium Phosphate
BMI: Body Mass Index
BMP: Bone Morphogenetic Protein
CBM: Cellular Bone Matrix
DBM: Demineralized Bone Matrix
DMSO: Dimethyl Sulfoxide
EGF: Endothelial Growth Factor
FFI: Foot Function Index
IBM: Integrative Bone Matrix
ICBG: Iliac Crest Bone Graft
MCS: Mental Component Score
PCS: Physical Component Score
PDGF: Platelet-Derived Growth Factor
PROM: Patient-Reported Outcome Measure
rhPDGF-BB: Recombinant human Platelet-Derived Growth Factor BB homodimer
SD: Standard Deviation
SF-12: Short Form-12
TGFβ3: Transforming Growth Factor-β3
VAS: Visual Analog Scale
VBM: Viable Bone Matrix
VEGF: Vascular Endothelial Growth Factor
